# Review of Current Cell-Penetrating Antibody Developments for HIV-1 Therapy

**DOI:** 10.3390/molecules23020335

**Published:** 2018-02-06

**Authors:** Muhamad Alif Che Nordin, Sin-Yeang Teow

**Affiliations:** 1 Kulliyyah of Medicine and Health Sciences (KMHS), Kolej Universiti INSANIAH, 09300 Kuala Ketil, Kedah, Malaysia; alif.nordin@outlook.com; 2 Sunway Institute for Healthcare Development (SIHD), School of Healthcare and Medical Sciences (SHMS), Sunway University, Jalan Universiti, Bandar Sunway, 47500 Subang Jaya, Selangor Darul Ehsan, Malaysia

**Keywords:** HIV-1, cell-penetrating, antibody, intracellular protein, therapy

## Abstract

The discovery of highly active antiretroviral therapy (HAART) in 1996 has significantly reduced the global mortality and morbidity caused by the acquired immunodeficiency syndrome (AIDS). However, the therapeutic strategy of HAART that targets multiple viral proteins may render off-target toxicity and more importantly results in drug-resistant escape mutants. These have been the main challenges for HAART and refinement of this therapeutic strategy is urgently needed. Antibody-mediated treatments are emerging therapeutic modalities for various diseases. Most therapeutic antibodies have been approved by Food and Drug Administration (FDA) mainly for targeting cancers. Previous studies have also demonstrated the promising effect of therapeutic antibodies against HIV-1, but there are several limitations in this therapy, particularly when the viral targets are intracellular proteins. The conventional antibodies do not cross the cell membrane, hence, the pathogenic intracellular proteins cannot be targeted with this classical therapeutic approach. Over the years, the advancement of antibody engineering has permitted the therapeutic antibodies to comprehensively target both extra- and intra-cellular proteins in various infections and diseases. This review aims to update on the current progress in the development of antibody-based treatment against intracellular targets in HIV-1 infection. We also attempt to highlight the challenges and limitations in the development of antibody-based therapeutic modalities against HIV-1.

## 1. Introduction

The development of HAART has significantly reduced AIDS-related death cases [1]. This strategy targets multiple viral proteins or processes (e.g., viral entry, reverse transcription, integration, transcription, and virus assembly and production) that are superior to monotherapy which targets a single protein (e.g., glycoprotein, reverse transcriptase, protease, etc.) at a time [2]. HAART has successfully reduced the viral load to <50 copies and replenished the number of CD4+ T-cells, thereby improving survival while reducing HIV infectivity [3]. Despite success seen in HAART, HIV infection has now turned into a chronic condition due to long-term use of HAART. A number of conditions has been reported with long-term exposure to HAART such as lipid metabolism disturbance that can predispose the patient to a number of cardiovascular problems [4]. In short, there are several challenges that limit HAART such as: (a) low specificity; (b) high toxicity; (c) high cost; and (d) promoting the development of escape mutants [5]. Furthermore, HAART drugs are unable to address the problem with HIV reservoir. Latently infected resting T cells provide a stable reservoir for HIV-1 despite strict adherence to HAART [6,7,8]. The risk of reactivation of these latent reservoirs are high when there is immune stimulation [7] and interruption of the HAART treatment [8]. Although the ultimate focus of HIV research is to achieve a sterilizing cure for HIV, we are still far from it. Thus, current research focus should be on maximizing the functional cure for HIV, without compromising the patient’s quality of life with debilitating burden of chronic illnesses.

Antibody-mediated therapy has emerged as a promising therapeutic strategy against various infectious diseases and cancers to counteract the abovementioned limitations. In the past 15 years, considerable attention has been drawn to the development of monoclonal antibodies (mAbs) against cancers [9,10]. Similarly, several studies have demonstrated the potential antiviral activities of antibodies against HIV-1 [11,12,13] while most of them are yet to be approved by FDA. Since full-length antibodies typically have the approximate size of 150 kDa, they have restricted access to the inner compartments of the cells for intracellular proteins, resulting in the confined therapeutic targeting against surface or extracellular protein [14,15]. Cumulative findings have shown essential roles of several intracellular proteins in the viral replication and infectivity such as p24/capsid protein [16,17], and other accessory proteins (e.g., Nef, Tat, and Rev) [18]. HIV-1 Nef and p24 capsid proteins have gained interest as promising targets for potential anti-HIV treatment in recent years [19,20,21]. The debut of heterologous expression of a biologically active recombinant HIV-1 p24 [22] and Nef [23] has propelled the development of potent cell-penetrating antibodies to inhibit HIV-1 infected T-lymphocytic cells line and PBMCs [24,25]. Similarly, HIV-1 Tat and Rev have also been targeted by cell-penetrating antibodies and promising antiviral effects have been observed [26,27,28,29]. Hence, blocking the biological activities of these proteins could be promising antiviral strategies. Several modifications/engineering have permitted the antibodies to gain access into the cells and specially target the intracellular protein of interest. These modifications are mainly performed through two methods, chemical or genetic/molecular [14,15]. This review aims to provide insights into the application of therapeutic cell-penetrating antibodies in combating HIV-1. We also attempt to discuss the potential limitations and challenges during the development of cell-penetrating therapeutic antibodies against HIV-1.

## 2. Targeting HIV-1 Intracellular Proteins as Therapeutic Targets

This section describes the important functions of viral proteins essential for viral replication and the accessory proteins that play roles in virus dissemination, which make them ideal targets for the development of various antiviral treatments. The therapeutic antibodies targeting these intracellular proteins will be discussed individually in Section 3.

### 2.1. Capsid Proteins (CA), p24

**Figure 1:**
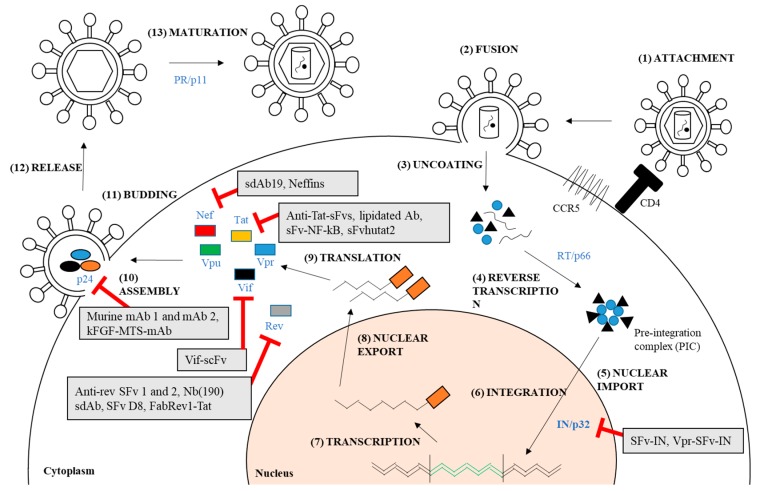
HIV-1 replication cycle and the antiviral targets for antibody-mediated treatment which have been previously reported. (1) The virus attaches to the cell by engaging the receptor CD4 and co-receptor CC-chemokine receptor 5 (CCR5); (2) This leads to the fusion of virus and cell membrane; (3) The capsid protein uncoats and releases the viral materials; (4) This allows reverse transcription that involves reverse transcriptase (RT) to take place; (5) This also yields pre-integration complex (PIC) which is then imported into the nucleus; (6) Integrated provirus is formed by integrase (IN) (can be targeted by SFv-IN and Vpr-SFv-IN); (7) Proviral transcription mediated by host RNA polymerase, takes place in the nucleus; (8) The mRNA is exported out from the nucleus; (9) Viral proteins are produced in the cytoplasm (i.e., Nef, Tat, Vpu, Vpr, Vif, Rev, and p24); The proteins that can be targeted by therapeutic antibodies are depicted in the diagram; (10) The viral RNA and proteins assemble on the cell membrane and repackage into a viral particle. This step is facilitated by p24 that can be targeted by murine mAb 1 and 2, and kFGF-MTS-mAb; (11 & 12) The viral particle buds and releases from the cells; (13) The virus then matures into an infectious viral particle mediated by proteases (PR).

HIV-1 Gag precursor (Pr55Gag) is composed of N-terminal matrix (MA/p17), capsid (CA/p24), nucleocapsid (NC/p7), C-terminal p6, and spacer peptides Sp1 and Sp2 [30]. Following a sequential proteolytic process, Gag is cleaved by a protease into CA and NC, which subsequently assemble to form the central core containing the viral RNA genome [31]. The presence of these proteins is abundant; a mature HIV-1 core has approximately 1500 CA monomers and up to 5000 Gag molecules in the immature core [32]. HIV-1 CA plays essential roles in early and late stages of viral replication [20]. During assembly, the cone-shaped capsids encapsulate the viral genome and upon membrane fusion, the capsids disassemble to release the genetic materials for replication in the cells [33]. In the late stage, p24 molecules assemble and re-package the viral genome to form viral particles [34]. These virions are released from the infected cells for dissemination (Figure 1). Targeting and sequestering the CA protein during these important steps can efficiently block the completion of viral life cycle. The potential of developing CA into a therapeutic target for HIV therapy has been recently reviewed [20,21,35]. Various therapeutic compounds have been found to target the CA and inhibit HIV-1 replication and/or infectivity. These include C1 [20], Ebselen [36], benzodiazepines (BD3) and benzimidazoles (BM4) [37], I-XW-053 [38], PF74 [39], Bevirimat (BVM) [40,41], CAP-1 [42], CAI [43], NYAD-1 and NYAD-13 [44], and BI-1 [45]. Among these molecules, Ebselen and I-XW-053 targeted HIV-1 early pre-integration or capsid uncoating process, while PF74, C1, CAP-1, CAI, BVM, NYAD-1, NYAD-13, BD3, and BM4 targeted HIV-1 maturation to prevent capsid assembly and virus release to new cells. Cumulative findings demonstrate that the majority of current CA-targeting compounds target the virus maturation step at its late-stage of the replication cycle involving the capsid assembly and mature virus formation [20,30,46]. Furthermore, antibodies targeting CA in HIV-1-infected cells are potent enough to exhibit a promising antiviral effect [24]. Development of an anti-HIV single-chain antibody (scFvs/sFvs) format that has higher cell-permeating capacity may also improve the HIV-1 targeting capabilities thereby enhance the antiviral action.

### 2.2. Nef

HIV-1 Nef is a viral accessory protein. Nef proteins are not directly involved in the viral replication, but they play essential roles in viral pathogenesis at both early and late phases. The functions of Nef in disease progression has been extensively reviewed [47,48,49,50]. Nef is a pathogenic factor that is expressed early in the viral life cycle [48]. The primary roles of Nef in the viral pathogenesis include CD4 downregulation, MHC class-I downregulation, CD28 downregulation, T-cell activation, and CD8αβ downregulation [51]. Nef proteins perpetuate the HIV-1-derived exosome secretion and contribute to the exosome-mediated disease pathogenesis [52,53]. In animal models, Nef-deleted mutants exhibited up to 40% reduction of infection rates and did not progress to AIDS compared to wild-type HIV-1, has paved a potential target for therapeutics [47]. In the early phase, Nef regulates the fusion properties of the cells [54] while in the late phase, Nef is responsible in stimulating viral reverse transcription, hence increasing the number of infectious virions and enhancing the infection of new cells [55]. The therapeutic blocking of Nef-mediated pathogenesis is expected to inhibit HIV-1 replication, infectivity, and viral spreading. Bouchet and co-workers have demonstrated that the phage display-derived antibodies targeting HIV-1 Nef are able to inhibit a cascade of Nef-mediated pathogenic effects (Table 1) [56,57]. Other Nef-targeting molecules such as diphenylpyrazolo or B9 [58], batzelladine and crambescidin analogs [59], and dihydrobenzo-1,4-dioxin-substituted analog of 2-quinoxalinyl-3-aminobenzene-sulfonamide (DQBS) [60] have also shown promising inhibition against Nef-dependent HIV-1 activities.

### 2.3. Tat

HIV-1 Tat is known to play a pivotal role in HIV-1 replication and infectivity by mainly activating the transcription from the viral long terminal repeat (LTR) promoter by binding to the transactivation-responsive region (TAR) hairpin in the viral RNA transcript [74]. Das and colleagues showed that Tat inactivation inhibited the HIV-1 replication, however, the viruses were able to replicate without Tat in SupT1 T cells when the U3 sequences were substituted with nonrelated promoter elements [75]. It has also been shown that the DDX3 interacts with HIV-1 Tat to facilitate HIV-1 mRNA translation, and knockdown of DDX3 inhibited Tat-dependent HIV-1 production [76]. As HIV-1 Tat and the Tat-binding TAR RNA play important roles in HIV-1 transcription, they are potential therapeutic targets for antiviral treatment [75,77,78]. Hamy and colleagues showed that the stilbene derivative CGA137053 inhibited HIV-1 by binding to Tat protein [79]. Mhashilkar and coworkers have developed several phage-display-derived anti-Tat antibodies that efficiently inhibited HIV-1 replication and infectivity (Table 1) [26,27,71]. Plant-derived compounds such as triptolide and curcumin have also been shown to reduced Tat-mediated LTR promoter transactivation and HIV-1 production by promoting the degradation of Tat protein. Of note, triptolide targeted Tat at the stage of viral gene transcription while curcumin does not affect Tat gene transcription [80,81]. Lacombe and colleagues also showed that spironolactone (SP), an aldosterone antagonist targeted Tat-dependent transcription and resulted in potential inhibition of HIV-1 and HIV-2 infections [82]. On the other hand, TAR RNA has been targeted by cyclic peptidomimetic L50 which subsequently blocked Tat-TAR interactions followed by the inhibition of HIV-1 transcription and replication [83]. Generally, Tat is known as an intracellular protein mainly due its transduction domain that has been previously shown to transport a variety of cargos across the cells [84,85]. On the other hand, there are also pieces of evidence showing that this protein could be released extracellularly and play important roles in viral infection [86,87]. Since the localization of this protein determines the efficiency of Tat-specific antiviral treatment, particularly in anti-Tat antibody-mediated treatment, further investigations are worthwhile to study the mechanism of Tat localization, followed by their effects on the antiviral treatment.

### 2.4. Rev

HIV-1 Rev is also one of the accessory proteins and has been known to be essential for both early and late phases of virus replication cycles. Rev is a transactivating protein that interacts with Rev response element (RRE) which is encoded by *env* gene [88]. In the early phase, HIV-1 Rev regulates the integration frequency to prevent cellular superinfection while in the late phase, it enhances the expression of viral proteins [89,90]. Blocking the activities of Rev and the interaction between Rev and RRE, have resulted in marked reduction of HIV-1 replication and infectivity. For instances, phage display-generated anti-Rev antibodies have shown potent antiviral effects against various HIV-1 strains (Table 1) [28,29,70]. Other Rev-targeting molecules that possess therapeutic activities against HIV-1 include PKF050-638 [91], SUMO-1 heptapeptide protein transduction domain for binding Rev (SHPR) [92], 3-amino-5-ethyl-4,6-dimethylthieno[2,3-*b*]pyridine-2-carboxamide and 4-amino-6-methoxy-2-(trifluoromethyl)-3-quinolinecarbonitrile (termed as 103833 and 104366) [93], 8-azaguanine and 2-(2-(5-nitro-2-thienyl)vinyl)quinoline (termed as 5350150) [94], and pyrimidin-7-amine, biphenylcarboxamide, and benzohydrazide, (designated as 791, 833 and 892) [95]. 

### 2.5. Other Proteins

In addition to the abovementioned target proteins, other intracellular proteins such as HIV-1 reverse transcriptase (RT)/p66, integrase (IN)/p32, protease (PR)/p11, nucleocapsid (NC)/p7, and matrix protein (MA)/p17 are among the promising candidate targets for antiviral treatment as reported in previous reviews [2,96]. Limited studies have also shown antiviral effects by targeting other accessory proteins such as Vif, Vpr, and Vpu [97,98,99]. While targeting these HIV-1 proteins resulted in marked HIV-1 inhibition, the viruses have a tendency to resist or escape the drug or inhibitor treatments, including HAART targets such as RT, IN, PR, etc. and other non-HAART target proteins such as Nef, p24, and so on [100,101,102]. For examples, Zhou and coworkers reported the development of a mutant virus that was resistant to the capsid-targeting PF74 inhibitor treatment and resulted in impaired viral replication in target cells [103]. Similarly, Shuck-Lee and colleagues demonstrated resistant viral variants towards HIV-1 Rev inhibitors-103833 and 104366 that possess two single-point mutations in the RRE [104]. Furthermore, the mutations in HIV-1 Vpu and Env have resulted in HIV-1 escape from interferon-induced transmembrane (IFITM1) protein inhibition [105]. Nef has also been shown to evade the restricted HIV-1 replication of both multipass transmembrane proteins serine incorporator 3 (SERINC3) and SERINC5 [106,107]. These findings highlight the importance of targeting viral proteins that evade the HIV-1 restrictions such as Vpu, Env, and Nef. Due to limited data available, the risk of emergence of escape mutants from intracellular viral protein-targeting antibodies is yet to be confirmed. Further investigations are needed to evaluate the risk of emerging viral resistance posed by this strategy. In the following section, we will focus and discuss the intracellular proteins HIV-1 p24, Nef, Tat, Rev, and IN in which antibody-mediated antiviral therapies targeting these proteins have previously shown excellent therapeutic values.

## 3. Development of Cell-Penetrating Antiviral Antibodies

**Figure 2:**
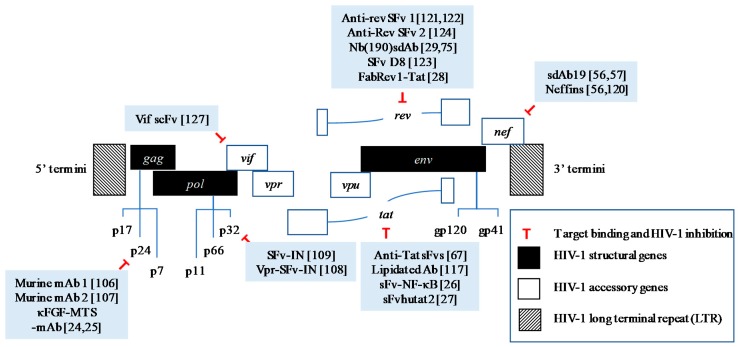
HIV-1 genes and their encoded proteins targeted by full-length antibodies or intracellular antibody fragments with promising therapeutic activities.

HIV intracellular proteins have garnered tremendous interest as important targets for development of anti-HIV-1 treatment. Several compounds targeting these proteins have shown promising results in inhibiting the viral replication and infectivity and are currently undergoing clinical trials [108,109,110]. A HIV capsid inhibitor, GS-CA1, has shown promising antiviral action against various HIV-1 strains in early laboratory and animal studies with minimal toxicity. This compound is expected to enter Phase I clinical trials in 2018 [111]. Continuous efforts are in progress in development of new inhibitors targeting HIV PR, RT, and IN [109]. Similarly, antibody-based therapies against HIV-1 intracellular proteins have recently drawn attention since the discovery of potent broad-neutralizing antibodies (bNAbs) in combating various viral infections such as Dengue virus (DV) [112], Hepatitis C [113], and HIV-1 [114]. Furthermore, antibody-mediated therapy possesses certain advantages over the chemical- or molecule-based therapies including well-tolerated safety and pharmacokinetics [115], longer half-lives [115,116], ability to opsonize viral particles [116], and virus killing by immune cells [116]. In this section, we will discuss the current progress of antibody-mediated treatment that are targeting intracellular HIV-1 proteins, both structural and accessory proteins. The therapeutic potentials of targeting these proteins are shown in Figure 1 and Figure 2.

### 3.1. Targeting the Viral Proteins Essential for Replication

So far, HIV-1 p24 and IN are the only intracellular proteins targeted by antibody-mediated therapy due to their direct involvement in viral replication (See Table 1). In 1992, Franke and colleagues developed a therapeutic full-length anti-p24 murine antibody that inhibited the release of reverse-transcriptase (RT)-active virus particles from HIV-1-infected cell lines and primary T-cells up to 60% [64]. They also successfully demonstrated the magnitude of the HIV-1 inhibition by delaying the viral spread for six days in in vitro cultures [64]. Since full-length conventional mAbs were used, it is presumed that the antiviral activities are merely implicated by the small percentage of antibodies that have successfully gained access into cells. Another explanation is that the anti-p24 mAbs targeted the extracellular p24 on the cell surface to exert the antiviral effect, which is unlikely. If the former speculation is true, the antiviral activity can be improved by enhancing the antibody’s cellular penetrance.

Ali et al. tested this idea by chemically conjugating the native anti-p24 mAbs with a cell-penetrating peptide, κFGF-MTS, to improve the cell-internalizing capacity of mAbs [24]. The conjugated κFGF-MTS-mAbs showed inhibition of the HIV-1 replication by 73% and 49% in T-cells and PBMCs, respectively, compared to the parallel native or unconjugated mAbs treatment which exhibited minimal to non-existent reduction in the viral replication. Furthermore, the conjugated antibody has also shown a modest antiviral effect (20–40%) in vitro against monocytes or macrophages in vitro [25]. The modest HIV-1 antiviral effects exhibited by the internalized antibody compared to the antiviral compounds showed that more rigorous work needed to be done to improve the efficacy of internalized antibody into a therapy. Nevertheless, the potential of adopting these antibodies into an adjunct therapy with current HAART treatment may prove beneficial [117]. This increasing attention has been drawn against p24 due to its indispensable role in viral replication and spreading [16,20,21].

Meanwhile, continuous efforts are in progress to explore the potential of antibody therapy development against other essential viral proteins such as MA, RT, IN, and PR [2,96,109]. There are several publications on the HIV-1 IN by intracellular antibodies for the past two decades over its role in mitigating the HIV-1 viral replication and infectivity [61,62]. However, none described the other issues such as in vivo HIV-1 inhibition, toxicity, stability, and bioavailability in blood.

### 3.2. Targeting the Accessory Proteins

A relatively higher number of publications described the cell-penetrating antibodies targeting HIV-1 accessory proteins compared to other viral components because they are mostly intracellular proteins (Table 1). Although these proteins are not directly involved in HIV-1 replication, their roles in viral spreading are nonetheless indispensable. 

One of the earliest intracellular proteins that were targeted for antiviral therapy development was Tat protein. The Tat protein has a cell-internalizing capacity as presented in various cell types [118] as well as tissues [119]. Since the discovery of this unique characteristic, it is useful in various applications such as intracellular delivery of molecule or drug [120], photodynamic therapy [121], and molecular imaging of intracellular proteins [122]. Tat protein is also used as a cell-penetrating peptides (CPPs) or protein transduction domains (PTDs), which also can be used to construct cell-penetrating antibodies [24,28]. In HIV-1 pathogenesis, the Tat protein is also one of the virulence factor for HIV-1 spreading despite not directly involved in the viral replication [123,124]. Mhashilkar and colleagues have developed therapeutic intracellular single-chain (sFvs/scFvs) format antibodies against Tat using phage-display method. The antibody-expressing non-infected T-lymphocytes have shown resistance and protection from the HIV-1 infection [71]. The antibody was then modified with an addition of NF-κB antagonists in which the conjugate has successfully prolonged HIV-1 inhibition up to 45 days [26]. To evaluate its clinical use, the humanized version of anti-Tat antibody (sFvhutat2) was constructed and its antiviral effect has been shown in primary HIV-1 isolate-challenged PBMCs [27]. Meanwhile, another group has chemically modified the anti-Tat antibody by lipidation and these modified antibodies inhibited various HIV-1 isolates up to 85% [72].

The pathogenic roles of HIV-1 Nef protein in the viral replication and spreading are well described [125,126]. Bouchet and co-workers developed a single-domain antibody fragment, sdAb19 that has a high binding affinity towards intracellular Nef. The antibody–antigen interaction capable of blocking several Nef-mediated effects in the responder cells including inhibition of CD4 down-modulation, inhibition of p21-activated kinase 2 interaction and actin remodeling, inhibition of viral infectivity and replication in PBMCs, and prevention of thymic CD4 T-cell maturation and peripheral CD4 T-cell activation in vivo [57]. The same group then conjugated the sdAb19 with modified SH3 domains (also termed Neffin) which resulted in more enhanced anti-Nef activities including inhibition of both CD4 and MHC-I down-modulation, and inhibition of Nef pathogenic effects in both T-cells and macrophages, inhibition of virus infectivity and replication [65]. The crystal structure and structure–function relationship of antibody binding has also been previously determined [56]. Collective findings suggest that Nef is a promising target for the development of HIV-1 inhibitors. 

In addition to HIV-1 Tat and Nef, targeting Rev protein by intracellular antibodies has also shown promises in antiviral therapy development. About twenty years ago, Duan and colleagues developed an anti-Rev single-chain antibody [66,67]. When expressed intracellularly in human T-cells, long-term inhibition of HIV-1 replication was observed for up to several months. Similarly, potent antiviral activities by the phage-display-derived anti-Rev SFvs were also respectively demonstrated in PBMCs [68] and primary monocytes [69]. On the other hand, Vercruysse and group developed an anti-Rev single-domain intrabody, Nb190, which potently interrupted the assembly of Rev multimers [70]. The blockade of assembly implicated in the suppressed HIV-1 RNA expression was followed by the inhibited viral replication when tested in HIV-1-infected cell lines. Further studies also demonstrated that Nb190 was able to inhibit a wide range of HIV-1 subtypes and groups due to the conserved targeting epitope of the antibody [29]. The binding site and interaction interface of Nb190-Rev have also been mapped to enhance the understanding of epitope-function studies [70,127]. Interestingly, Zhuang et al showed the improved antiviral effect of anti-Rev Fab in PBMCs when the antibody was conjugated to HIV-1 Tat [28]. This may be due to the improved cell internalization of the antibody by the cell-permeating Tat peptide, which resulted in enhanced interaction and antiviral action. HIV-1 Vif has also been targeted by intracellular antibodies generated from rabbit immunization (Table 1) [73]. The Vif scFvs were shown to accumulate in the cytoplasm and efficiently inhibited viral reverse transcription and replication in various HIV-1-challenged cell lines such as Jurkat, SupT1, CEM, H9, U38, and primary PBMCs.

## 4. Challenges and Future Directions

### 4.1. Technologies in Cell-Penetrating Antibody Development

The full potential of a conventional antibodies as therapeutic molecules is hampered by its relatively larger size compared to other smaller formats which has limited their targets to extracellular only. Strategies have been employed to improve their cell permeability and to subsequently allow the complete targeting and blocking of intracellular proteins. Currently, the phage display library technique is the most commonly used molecular method in constructing cell-penetrating versions of antibodies. The end products are either in Fabs, sFvs or sdAbs formats which have approximate sizes of 50, 25 and 15 kDa, respectively (Table 1). The Fc region of these antibodies is removed which contributes to the significant reduction of total size [128,129] although its removal may lead to the loss of antiviral activity [129]. Hence, several methods have been developed to make the antibodies cell-penetrable while conserving the Fc region that possesses functional activities [24,130]. These methods primarily require site-specific chemical or biological modifications that permit linkage with molecules that assist in cell permeability such as cell-penetrating peptides, polymers, nucleotides, and so on [131]. Although these techniques are cheaper, they are relatively laborious, less consistent, and the cell-penetrating potency largely varies depending on multiple factors such as temperature, pH, and chemical concentration [24,130]. In addition, the chemical linkage of conjugated antibodies is less stable during storage compared to genetically constructed antibodies [25].

With the advent of phage-display technology, high-affinity antibodies can be generated with only a specific set of primers thereby allowing versatility of the construction of small antibody fragments with desired genetic modifications (e.g., conjugation with fusion partner, simian virus 40 (SV40), adaptation into SV40 delivery system, and modification with other function domains) (Table 2) [128]. For instance, the anti-Rev SFv was cloned into a SV40 expression vector forming SV(Aw) to improve the efficiency of intracellular delivery. Another novel method, the engineering of an anti-IN scFv gene in an armed SV40 viral vector has bypassed the need for laborious and other limitations of delivery of actual antibody into the target cell cytoplasm. Such a gene therapy method represents a useful new class of anti-retroviral agents [61]. The same group also genetically fused the anti-integrase SFv (SFv-IN) with HIV-1 Vpr by molecular cloning in which the end-product Vpr-SFv-IN resulted in a marked decrease of virion infectivity [62]. Another successful example was the development of Neffins, a fusion of anti-Nef sdAb with SH3 domain which has inhibited a cascade of Nef-mediated pathogenic effects in HIV-1-infected cells [65]. Despite its excellent cell permeability, the main challenge of adopting these antibodies for therapeutic use is the questionable stability and bioavailability compared to full-length antibodies [129]. The antibody bioavailability is expected to deteriorate when the antibodies are administered in vivo in the animal studies even before entering clinical trials [132,133]. To this, several strategies have been employed to extend the half-life of these antibodies, including the fusion to immunoglobin-binding domains [134], albumin binding [135], polymer [136] and so on.

Moreover, other arising issues of application of small-fragment antibodies as an antiviral therapy include the production cost and potential cytotoxicity [129]. The high production cost of therapeutic antibodies is notorious due to the need of generating them in a host system by animal immunization. The advances in molecular methods have emerged and the antibodies can now be generated using bacteriophage by phage display. This technique does not only reduce the production cost but also allows other genetic manipulations and versatility such as affinity selection and maturation, antibody binding region sequencing, and fusion with various genes [128]. Apparently, this method has several advantages over the conventional method of antibody production. Although the toxicity of antibody-mediated treatment is less critical than the molecule- and chemical-based inhibitor treatment, several reports have demonstrated the toxicity of therapeutic antibodies by the target host immune system [137,138]. This has given rise to the development of humanized therapeutic antibodies to remove the non-human regions as much as possible, to minimize the unnecessary immunogenicity [139]. While current the molecular method allows the conjugation of various fusion partners to the therapeutic antibodies, attention should be drawn towards antibody engineering so that the end-products do not trigger an immune response, hence resulting in unnecessary cytotoxic effects.

### 4.2. Viral Resistance and Escape Mutants

The rise of HIV-1 resistance towards HAART is a global issue [140]. Current antibody strategies focus on the HIV-1 extracellular targets and often fail to provide sufficient viral load suppression in vivo due to rapid viral mechanisms, thereby limiting its potential to be developed into an effective therapy [119]. With proper modifications to the antibody, antibody treatment against HIV-1 intracellular targets are now made available. However, due to limited data available, more work is needed to be carried out to evaluate the risk of emerging viral resistance posed by the internalized antiviral antibodies. Interestingly, it has been reported that the antibodies are effective against various resistant HIV-1 variants [11,141,142]. Recently, efficient broadly neutralizing antibodies (bnAbs) against a wide range of HIV-1 strains have shown promising potentials in HIV-1 therapy and the clinical trials are in progress [114,143,144]. Although the development of escape mutants against antibody-mediated therapy is believed to be less common than the antiviral drug-resistant variants, continuous efforts are required to prevent the development of antibody-resistant HIV-1 mutants. In fact, there have been several studies suggesting viral resistance against antibody-mediated therapies. For instances, Manrique and colleagues have shown the viral escape from membrane-proximal external region (MPER)-specific MAbs in vitro, particularly in 2G12, but in general, the threshold of resistance evolution is extremely high which showed promises in targeting MPER by antibody-based treatment [143]. On the other hand, Wibmer and group showed the development of HIV-1 variants which escaped from gp120 V2- and CD4 binding site-targeting bnAbs in the patient plasmas, however, the patient immune system was able to generate multiple bnAbs in response to the emerging evolution of HIV-1 variants to combat the infection [141]. Interestingly, a mathematical framework termed as mutant selection window (MSW) has been recently developed to determine efficacy of anti-HIV-1 bnAbs in both free-virus infection and cell–cell transmission, and to prevent mutant selection [144]. More importantly, the outcome of the study highlighted the importance of combination therapy that involves multiple antibodies targeting different epitopes in the efforts of reducing the incidence of viral resistance and to use antibodies for long-term treatment. In fact, there have been several efficacious combination therapies against HIV-1 that have shown promises in the past [145,146].

### 4.3. Future Directions

Although HAART remains the standard therapeutic intervention to control HIV-1 infection, the treatment is unable to clear the virus and requires life-long administration which may eventually lead to drug resistance [58]. Antibody-mediated therapies minimize viral resistance in addition to their downstream functional properties that can trigger the immune system to collectively combat HIV-1 infection. For example, enhanced clearance of intracellular viruses and/or viral proteins can be made by directing the cytosolic antibody-bound viral particles to proteosomal degradation via cytosolic IgG receptor, tripartite motif-containing 21 (TRIM21) [147]. The cytosolic antibody-bound viruses or viral particles are bound by the TRIM21 via its terminal PRYSPRY domain at sub-nanomolar binding affinity, and leads to valosin-containing protein (VCP)-dependent degradation in a process known as antibody-dependent intracellular neutralization (ADIN) [148,149]. Moreover, TRIM21 also activates three immune pathways (NF-κB, AP-1 and IRF family) which support pro-inflammatory cytokine production, modulation of cell-surface ligands, and adoption of an antiviral state [149,150]. TRIM21 is anticipated to boost the potency of antibodies targeting intra-cellular proteins in HIV/AIDS treatment, however, further investigations are warranted.

In addition, current advancement in antibody engineering also allows the development of single antibody molecules that can target more than one viral antigen known as bi-, tri-, multi-specific antibodies, these may contribute to future antibody therapy compared to the use of multiple single-target antibodies [142,151,152]. These attributes of targeting intracellular HIV proteins by cell-penetrating antibodies could comprehensively target and clear both extra- and intra-cellular proteins and overcome the limitation that was previously confined to targeting cell surface proteins. This may have high potential in shifting the current paradigm of inhibitor-based HIV-1 treatment. Current findings highlight that targeting multiple viral targets (e.g., combination treatment) is the most efficacious strategy to control viral resistance and spreading [144,145,146]. Hence, the future of HIV therapy may adopt multiple therapeutic antibodies to provide a full-range of antiviral effects as an adjunct therapy to conventional HAART drugs [153]. 

While HIV-1 infection is still prevalent in developing countries, the advancement of technology plays an important role in addressing these problems to bring affordable therapy into primary care in the most remote settings. Other limitations of antibody-mediated therapies include off-target toxicities, poor bioavailability and stability, high renal clearance rate, and high individual-to-individual variation as mentioned above. Continuous efforts in discovering novel and robust viral targets must be continued to counter-act newly emerging viral resistance. 

## 5. Conclusions

HIV-specific cell-penetrating antibodies are more effective than small molecule inhibitors. These therapeutic modalities can simultaneously target both extra- and intra-cellular targets, and efficiently block and neutralize HIV-1 activities, hence, resulting in a more comprehensive antiviral action. As highlighted in this review, several challenges remain to be addressed and advanced technologies are anticipated to kick in to improve the current antiviral strategies. Continuous efforts are needed in seeking novel antibody-based therapies against HIV-1 and combination therapy is one of the promising approaches based on the current findings to control the emerging resistant HIV-1 variants and to prolong the feasibility of antibody-mediated HIV treatment.

## Figures and Tables

**Table 1:** Development of therapeutic antibodies against HIV-1 intracellular targets.

Target	Antibody	Conjugation/Engineering	Antiviral Effect
Integrase (IN)/p32	SFv-IN	SV40 as delivery system	Inhibition of HIV-1 replication and syncytium formation in human T-lymphoid cell, SupT1 [61]
	Vpr-SFv-IN	Phage-display and fusion to Vpr protein	Inhibition of HIV-1 replication in human T-lymphoid cell, SupT1 and reduction of virion infectivity [62]
Capsid (CA)/ p24	Murine anti-p24 Mabs	Native	Inhibition of active virus particles in up to 60% in HIV-1 infected cell lines or IL-2 stimulated T-cells [63]
	Murine anti-p24 Mabs	Native	Delay of HIV-1 spread for 6 days in in vitro cell culture [64]
	κFGF-MTS-anti-p24-mAbs	κFGF-MTS peptide chemical conjugation	Inhibition of HIV-1 replication up to 73% and 49% in T-cells and PBMCs respectively [24,25]
Nef	sdAb19	Phage-display	Inhibition of Nef-mediated CD4 down-regulation [56,57] Inhibition of p21-activated kinase 2 interaction and actin remodeling [57] Inhibition of viral infectivity and replication in PBMCs [57] Prevention of Nef-mediated thymic CD4 T-cell maturation and peripheral CD4 T-cell activation in vivo [57]
	Neffins	Phage-display and fusion to modified SH3 domains	Inhibition of CD4 and MHC-1 cell surface downregulation [56,65] Inhibition of all functions of Nef in both T-cells and macrophages [65]
Rev	Anti-Rev SFv	Phage-display	Inhibition of replication of various laboratory and primary clinical HIV-1 strains in long-term human T-cell lines for several months [66,67]
	SFv D8	Phage-display	Inhibition of HIV-1 production in human T-cell lines and PBMCs [68]
	Anti-Rev SFv	Phage-display	Inhibition of HIV-1 replication in chronically infected U1 promonocytic cell line, ACH-2 T-cell, and primary monocyte cultures [69]
	Nb(190) sdAb	Phage-display	Inhibition of replication of wide range of different HIV-1 subtypes [29,70]
	FabRev1-Tat	Phage-display and Tat peptide conjugation	Inhibition of viral replication of CCR5-tropic HIV-1 isolates in PBMCs [28]
Tat	Anti-Tat sFvs	Phage-display	Resistance of antibody-expressing lymphocytes to HIV-1 infection [71]
	Lipidated anti-Tat antibody	Lipidation chemical modification	Inhibition of HIV-1 replication of several HIV-1 isolates by 85% [72]
	Anti-Tat sFv with NF-κB antagonists	Phage-display	Longer inhibition of HIV-1 replication up to 45 days [26]
	sFvhutat2	Phage-display	Inhibition of HIV-1 replication in HxB2- and two syncytium-inducing (SI) primary isolates- challenged PBMCs [27]
Vif	Vif scFv	Phage-display	Inhibition of HIV-1 replication in HIV-1 infected primary cells and cell lines [73] Inhibition of completed reverse transcripts formation [73]

**Table 2:** Technologies of cell-penetrating antiviral antibodies development in the past twenty years.

Method	Details	Therapeutic Antibody
Genetic	Phage display	sFv [27]
	Cloning of SFv into SV40 expression vector	SV(Aw) [61]
	Phage display and fusion to Vpr by cloning	Vpr-SFv-IN [62]
	Ilama immunization and phage display	sdAb [57]
	Phage display and fusion to SH3 by cloning	sdAb-SH3 (Neffins) [65]
Chemical	Lipidation chemical modification	Lipidated antibody [72]
	Conjugation to κFGF-MTS cell-penetrating peptide	κFGF-MTS-mAbs [24,25]
Genetic & chemical	Phage display and conjugation to Tat cell-penetrating peptide	Fab-Tat [28]

## Abbreviations

ADIN	antibody-dependent intracellular neutralization
AIDS	acquired immunodeficiency syndrome
bnAb	broad-neutralizing antibody
CA	capsid
CD	cluster of differentiation
CPP	cell-penetrating peptide
Fabs	antibody fragment
Env	envelope
Fc	fragment crystallizable fragment
FDA	Food and Drug Administration
gp	glycoprotein
HAART	highly active antiretroviral therapy
HIV-1	Human immunodeficiency virus type-I
IFITM1	interferon-induced transmembrane
IN	Integrase
kDa	kilodalton
LTR	long terminal repeat
MA	Matrix protein
mAbs	monoclonal antibodies
MHC	major histocompatibility complex
MPER	membrane-proximal external region
MSW	mutant selection window
NC	nucleocapsid
NF-κB	Nuclear factor kappa B
PBMC	peripheral blood mononuclear cell
PR	protease
PTD	protein transduction domain
RNA	ribonucleic acid
RRE	Rev response element
RT	reverse transcriptase
scFvs/sFvs	Single-chain variable fragments
sdAb	single-domain antibody
SHPR	SUMO-1 heptapeptide protein transduction domain for binding Rev
SH3	Src homology-3
SP	spironolactone
SV40	Simian virus 40
TAR	transactivation-responsive region
TRIM21	tripartite motif-containing 21
VCP	valosin-containing protein
